# Placental membrane aging and HMGB1 signaling associated with human parturition

**DOI:** 10.18632/aging.100891

**Published:** 2016-02-04

**Authors:** Ramkumar Menon, Faranak Behnia, Jossimara Polettini, George R Saade, Judith Campisi, Michael Velarde

**Affiliations:** ^1^ Department of Obstetrics & Gynecology, The University of Texas Medical Branch at Galveston, Galveston, TX 77555-1062, USA; ^2^ Buck Institute for Research on Aging, Novato, CA 94945, USA; ^3^ Department of Cell & Molecular Biology, Lawrence Berkley National Laboratory, Berkeley, CA 94720, USA; ^4^ Institute of Biology, University of Philippines, Diliman, 1101 Quezon City, Philippines

**Keywords:** pregnancy, preterm birth, MAPK, SASP, DAMPs, inflammation, fetal membranes, amnion, chorion

## Abstract

Aging is associated with the onset of several diseases in various organ systems; however, different tissues may age differently, rendering some of them dysfunctional sooner than others. Placental membranes (fetal amniochorionic membranes) protect the fetus throughout pregnancy, but their longevity is limited to the duration of pregnancy. The age-associated dysfunction of these membranes is postulated to trigger parturition. Here, we investigated whether cellular senescence—the loss of cell division potential as a consequence of stress—is involved in placental membrane function at term. We show telomere reduction, p38 MAPK activation, increase in p21 expression, loss of lamin B1 loss, increase in SA-β-galactosidase, and senescence-associated secretory phenotype (SASP) gene expression in placental membranes after labor and delivery (term labor [TL]) compared to membranes prior to labor at term (term, not-in-labor [TNIL]). Exposing TNIL placental membranes to cigarette smoke extract, an oxidative stress inducer, also induced markers of cellular senescence similar to those in TL placental membranes. Bioinformatics analysis of differentially expressed SASP genes revealed HMGB1 signaling among the top pathways involved in labor. Further, we show that recombinant HMGB1 upregulates the expression of genes associated with parturition in myometrial cells. These data suggest that the natural physiologic aging of placental tissues is associated with cellular senescence and human parturition.

## INTRODUCTION

Aging is accompanied by a decline in physiological function and the deterioration of many tissues and organs. Hence, aging is strongly linked to the onset of diseases in late adulthood, such as Alzheimer's, Parkinson's, cardiovascular diseases, and musculo-skeletal degeneration [1]. However, some tissues in the same organism may have different life spans than others. The most striking example of this are the placental membranes (fetal membranes or amniochorionic membranes), which are the innermost lining of the uterine cavity [2]. This tissue has a normal life span of only 9 to 10 months. Placental membrane cells (amnion and chorion cells) multiply and fully develop by the twelfth week of gestation, providing protection to the growing fetus. The membranes serve as a mechanical and Immune barrier during pregnancy between the mother and the fetus. The function of placental membranes ends once the fetus reaches maturity and is ready for delivery at term. Another function of the placental membranes is likely to promote labor and delivery of the fetus and placenta. Evidence shows that placental membrane cells enter senescence prior to labor [3]. Senescence of placental membrane cells is hypothesized to increase intrauterine inflammation to promote human parturition.

Normal term human parturition is initiated when fetal organ systems are mature—typically after 37–40 weeks of gestation. Conventional theories of how parturition signals are initiated primarily invoke feto-maternal endocrine and immune changes in the intrauterine cavity, correlating with fetal growth and development [4-9]. Homeostatic imbalances produced by such changes produce an inflammatory overload that disrupts the maintenance of pregnancy, resulting in labor-related changes [7,10-16]. Nonetheless, the signature of these signals and their precise mechanism in initiating parturition are unclear.

Cellular senescence is a response to stress and other physiological signals that culminates in an essentially irreversible growth arrest [17]. Senescent cells typically have a persistently high expression of the cell cycle inhibitors p21^WAF1^ and/or p16^INK4A^ [18], increased activity of a lysosomal enzyme termed senescence-associated β-galactosidase (SA-β-gal) [19], loss of nuclear HMGB1 and its secretion into the extracellular milieu [20], and decreased expression of lamin B1 [21]. Senescent cells also secrete several proinflammatory factors, called senescence-associated secretory phenotype (SASP) [22]. We recently reported a new fetal-derived signaling mechanism whereby oxidative stress (OS) at term is accompanied by telomere shortening and cellular senescence of placental membrane (amniochorion) cells [3, 23-25]. During pregnancy, placental membranes undergo telomere shortening [26]. Telomeres shorten throughout gestation and are shortest at term labor (TL), which correlates with increased OS [26,27]. At term, OS builds in the intrauterine cavity due to increased fetal metabolic demands, reduced maternal supply of metabolic substrates, depletion of antioxidants, and increased fetal stretching of placental and uterine tissues [28-32]. OS at term can promote telomere attrition [25], inflammation, and loss of placental membrane function through activation of the stress-associated kinase, p38 MAPK [3, 23-25, 33, 34]. Placental membrane cells at term also express inflammation-associated genes, reminiscent of the SASP [3,35]. Because placental membrane cells are thought to enter senescence prior to labor, and senescent cells are observed in placental membranes at term, cellular senescence may be part of the initiating stimulus resulting in human parturition.

In a cell culture model of fetal amnion epithelial cells (AEC) established from placental membranes from term, not-in-labor (TNIL) tissues obtained after Cesarean deliveries, OS caused p38 MAPK-associated senescence, senescence-associated morphologic changes, and increased inflammatory cytokine production [24, 34]. This sterile inflammation caused by senescent cells was inhibited by a p38 MAPK inhibitor, confirming the role of p38 MAPK in stress-associated senescence activation in fetal membranes at term [25,34]. In addition to expressing a SASP, senescent fetal cells also release damage-associated molecular pattern (DAMPs) and alarmins [36] that can amplify fetal immune responses and contribute to sterile inflammation [3]. The 2 DAMPs produced by senescent placental membranes, high mobility group box 1 (HMGB1) and cell-free fetal (cff) telomere fragments, can accelerate placental membrane senescence in a feedback loop and increase sterile inflammation through a p38 MAPK-mediated pathway [23, 25].

Based on the data described above, it is likely that the transition from not-in-labor to labor status at term involves the senescence of placental membrane cells and production of SASP factors and DAMPs. However, many of the data generated used clinical specimens obtained after labor and delivery of the placenta, so it remains unclear whether senescence is a cause or an effect of labor and delivery. The impracticality of prospective placental membrane sampling to test this hypothesis makes its validation difficult in humans.

This study was undertaken to further validate senescence-related cellular and histological changes and SASP gene expression in human placental membranes from TNIL and TL tissue. To recapitulate and validate these findings in culture, we used placental membrane explants and primary amnion cells collected and cultured from TNIL placentas exposed to cigarette smoke extract (CSE). CSE is used in our study as an OS inducer for 2 major reasons: 1) cigarette smoking during pregnancy is associated with fetal leukocyte DNA damage, placental dysfunction, adverse pregnancy outcomes, and epigenetic programming associated with adult onset diseases, providing biologic relevance to its use [37-44] and 2) CSE-induced OS and placental membrane cell senescence is well characterized in our cell culture system [24, 34, 45-48]. In addition, we examined the effect of the DAMP HMGB1 in producing contractility-associated changes in the myometrium. Overall, our data show that cellular senescence can occur in utero, particularly in fetal tissues like placental (fetal/amniochorionic) membranes and that this normal physiologic event during pregnancy may play a role in promoting human parturition.

## RESULTS

### Increased cellular senescence in human placental membranes at TNIL vs TL

**Figure 1:**
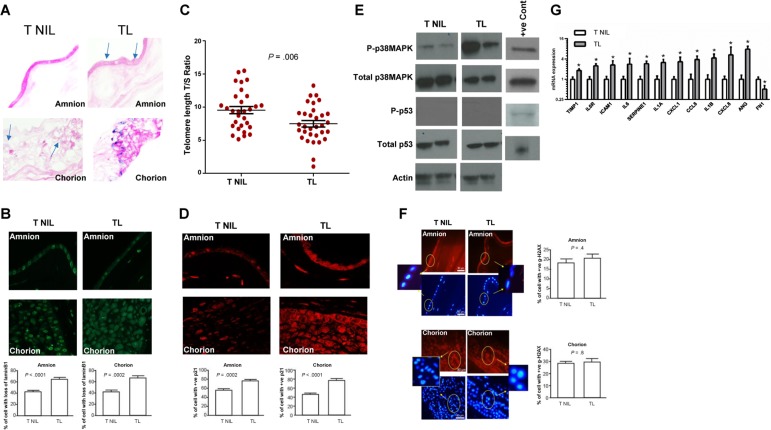
Cellular senescence in TL vs TNIL (**A**) Light microscopy of SA-β-gal staining: SA-β-gal stained cells (blue staining) from TL and TNIL (published data). The number of blue stained cells was significantly higher in both amnion and chorion from TL than TNIL (40x). (**B**) Microscopy of **lamin B1 staining**: Loss of lamin B1 is a sign of senescence. TL amnion (top) and chorion (bottom) had more loss of lamin B1 than TNIL (n=10) (40x) in each group. The percentage of cells with loss of lamin B1 was significantly higher in both compartments from TL than TNIL. Bar graphs represent the significant differences in the percentage of cells with loss of lamin B1. (**C**) Telomere length comparisons between TNIL and TL placental membranes samples are represented as T/S ratio. A significant decrease in telomere length was seen in TL samples compared to TNIL samples. (**D**) Microscopy of p21 immunostaining: p21 was not seen in our Western blot analysis; however, immunostaining of total p21 demonstrated increased staining in both amnion (top) and chorion (bottom) (40x) compartments of TL placental membranes but not in TNIL (n=10 in each group). The percentage of cells staining for p21 was significantly higher in both compartments from TL than from TNIL. Bar graphs represent the significant differences in the percentage of cells with p21 staining. (**E**) Representative blot images of P-p38 MAPK, total p38 MAPK, P-p53, and total p53 (from an n=10) in TNIL vs TL. P-p38 MAPK was intense in membranes from TL compared to TNIL. P-p53 was not seen in either TNIL or TL membranes, whereas total p53 was in both membranes. (**F**) Microscopy of Immunostaining for γ-H2AX: γ-H2AX or DNA damage foci indicate activation of DNA damage repair pathway. Neither amnion (top panel—red staining, below DAPI, inset shows γ-H2AX localization) nor chorion (bottom panel—red staining, below DAPI, inset shows γ-H2AX localization) from TNIL and TL showed any significant difference in the number of γ-H2AX stained cells (n=10). Bar graphs represent the percentage of cells with γ-H2AX staining. (**G**) qRT-PCR data of senescence and SASP-associated genes demonstrate significant changes in *TIMP1*, *IL6R*, *ICAM1*, *IL6*, *SERPINE1*, *IL1A*, *CXCL1*, *CCL8*, *IL1B*, *CXCL8*, *ANG*, and *FN1* between TL and TNIL placental membranes.

We collected placental membrane samples from normal term pregnancies with no prior history of pregnancy complications or any adverse events during the current pregnancy. Gestational ages were 39.52 ± 0.5 for TNIL and 39.89 ± 0.6951 (*P* = .2) for TL. Mean maternal ages were 25.70 ± 4.785 years for TNIL and 24.50 ± 6.311 years for TL (*P* = .6). Markers for senescent cells were evaluated in these TL and TNIL tissues. We observed an increased number of SA-β-gal positive cells in both the amnion and chorion of TL compared to TNIL (Figure 1A). We also observed a greater loss of lamin B1 in both the amnion and chorion layers from TL compared to TNIL amnion (*P* < .0001) and chorion (*P* < .0002) (Figure 1B). Because increased SA-β-gal activity and loss of lamin B1 are markers of cellular senescence, our data suggest that senescent cells accumulate in TL but not in TNIL.

Cellular senescence is often a consequence of stress. One prominent stressor is telomere shortening, which is associated with replicative senescence. We found the mean ratio of telomere fragments to single-copy gene number, a semi-quantitative estimation of telomere length, was significantly reduced in placental membrane samples from TL (n=30) compared to TNIL (n=30) (*P* = .006) (Figure 1C), consistent with the presence of senescent cells in TL placental membranes. Telomere shortening can also induce a persistent DNA damage response, resulting in elevated levels of cell cycle inhibitors, such as p21 [49, 50]. The number of p21 positive cells, detectable by immunostaining, was higher in both amnion (*P* = .0002) and chorion cells (*P* < .0001) in TL compared to TNIL (Figure 1D). Stress-associated p38 MAPK activation is often seen in placental membrane cells. We observed higher p38 MAPK activation in TL compared to TNIL tissue (Figure 1E). Surprisingly, p53, an upstream regulator of p21, remained low in both TNIL and TL (Figure 1E), which could reflect the low but persistent p53 signaling that is characteristic of senescent cells [51]. Moreover, no increase in DNA damage signaling was observed in amnion or chorion cells from TL and TNIL membranes, as determined by the similar number of cells that were positive for γ-H2AX foci (Figure 1F). These data suggest that the cellular senescence in TL tissues might be a consequence of only one or a few short telomeres; alternatively, it could be a consequence of other stressors present during TL.

Because senescent cells can also express and secrete SASP factors, we investigated their expressions. We measured the mRNA levels of genes encoding several SASP-associated factors in TNIL vs TL placental membranes (Figure 1G). Indeed, the expression of several SASP genes was upregulated in TL vs TNIL tissue, consistent with the increased number of senescent cells in TL vs TNIL tissues. Further, SASP gene expression is positively regulated by p38 MAPK [52], and Western blot analysis demonstrated p38 MAPK activation (P-p38 MAPK) in all TL samples compared to little or no activation in TNIL samples (Figure 1E). These results suggest that senescent cells in TL express a SASP, which includes proinflammatory factors [22].

### Increased cellular senescence in human placental membranes after CSE exposure

**Figure 2:**
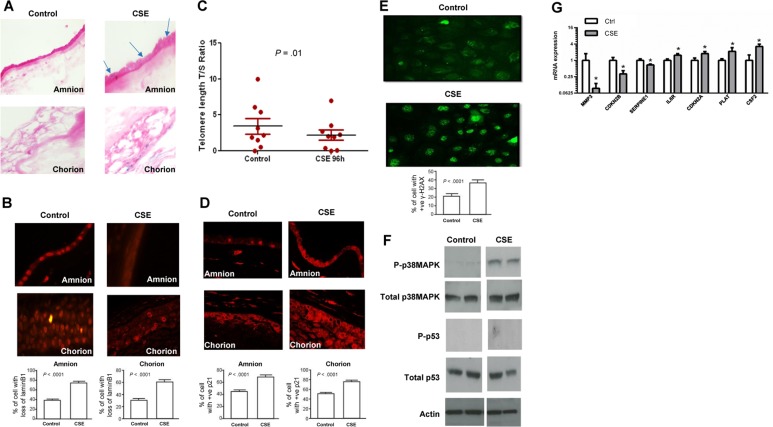
Cellular senescence in CSE-treated membranes (**A**) Light microscopy of SA-β-gal staining (blue staining). The number of blue stained cells was significantly higher in both amnion and chorion after CSE treatment than in untreated controls (40x) (n=10). (**B**Microscopy of lamin B1 staining: Similar to TL, lamin B1 loss was higher after CSE treatment in placental membrane amnion (top) and chorion (bottom) layers. Bar graphs represent the significant differences in loss of lamin B1. (**C**) Telomere length comparisons between untreated ‘control’ and CSE-treated amnion epithelial cells after 96 hours in culture. A significant decrease in telomere length was seen after CSE treatment of primary amnion epithelial cells derived from TNIL membranes in culture. (**D**) Microscopy of p21 immunostaining: Similar to TL, immunostained amnion (top) and chorion cells (bottom) were much higher after CSE treatment than untreated controls (n=10 in each group). Bar graphs represent the significant differences in the percentage of cells with p21 staining. **(E)** Microscopy of immunostaining for γ-H2AX: Primary amnion epithelial cells (AECs) from TNIL exposed to CSE (bottom panel) had a significantly higher number of γ-H2AX staining cells than untreated controls (n=5). Bar graphs represent the significant differences in γ-H2AX staining cells between the groups. (**F**) A representative blot of images of p38 MAPK and p53 in vitro. Similar to TL and TNIL, cultured placental membranes did not demonstrate any activation of p53 (n=10). Total p53 was seen in both CSE-treated and untreated controls, and no visible change in band intent sites were noted. (**G**) qRT-PCR data of senescence and SASP-associated genes demonstrate significant changes in *MMP3*, *CDKN2B*, *SERPINE1*, *IL6R*, *CDKN2A*, *PLAT*, and *CSF2* between controls and CSE-treated placental membranes.

To reproduce the above data in culture, primary fetal AECs or placental membrane cultures were treated with CSE for up to 24 hours and assessed for the markers described above. Telomere shortening and γ-H2AX foci are not seen in nondividing placental membrane organ explants, so we studied the other responses to OS in the intact membrane because it represents the in vivo status better than cells. We used TNIL placentas exposed to CSE to determine whether OS induces senescent phenotypes, SASP gene expression, and inflammation markers in this tissue. Similar to cells found in placental membranes at TL, primary amnion and chorion cells exposed to CSE showed an increased number of SA-β-gal positive cells (Figure 2A) and significant lamin B1 loss (*P* < .0001 for both amnion and chorion cells) (Figure 2B). These data suggest that CSE promotes cellular senescence in placental membranes.

We further assessed telomere length in primary AECs in response to CSE. CSE treatment of TNIL cultured amnion cells reduced telomere lengths after 96 hours compared to untreated control cells (*P* < .01) (Figure 2C), consistent with our reports showing that CSE generates 8-oxoguanine and amnion cell DNA damage, which is reversed by the antioxidant N-acetyl cysteine [34]. These data suggest that OS induced by CSE at term can accelerate telomere attrition, probably by targeting the guanine-rich telomere repeat sequences [22, 53, 54]. Similar to placental membranes at TL, CSE-treated placental membranes contained significantly more p21-positive cells than controls in both the amnion and chorion (both *P* < .0001) (Figure 2D). But unlike cells at TL, CSE treatment of fetal amnion cells from TNIL resulted in increased γ-H2AX foci compared to untreated controls (*P* < .001) (Figure 2E). This difference between clinical specimens and cell culture may suggest differences in the trigger of cellular senescence during normal labor compared to ex vivo cultures exposed to CSE, which can also trigger labor.

We also evaluated p38 MAPK and p53 activation (Figure 2F) and SASP expression (Figure 2G) in placental membranes exposed to CSE. When placental membranes from TNIL were placed in culture and exposed to CSE, p38 MAPK activation (as determined by phosphorylation; P-p38 MAPK) was similar to that of TL placental membranes (Figure 2F). Untreated placental membranes from TNIL (control) showed minimal activation of P-p38 MAPK (Figure 2F). In both TL and TNIL, p53 activation (P-p53) was not evident, consistent with our prior report showing p53 activation in primary amnion cells treated with etoposide but not CSE [25]. Interestingly, there was no overlap in SASP expression between our in vivo and cell culture studies (Figure 2G). Of course, cell cultures were treated with CSE for only 24 hours, which is sufficient to cause p38 MAPK activation but might not be sufficient for full establishment of the SASP, which generally takes several days [22]. Unfortunately, due to technical difficulties in keeping the membranes treated with CSE alive for several days, we were not able to demonstrate the same high expression of SASP factors as we did with membranes analyzed immediately after isolation from human subjects.

### IPA analysis reveals inflammation and HMGB1 signaling in vivo and in vitro

**Figure 3:**
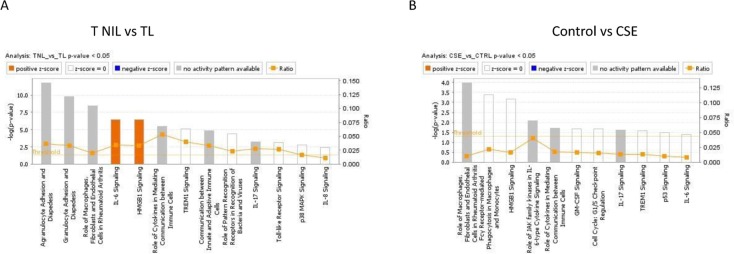
Canonical pathways identified by IPA analysis (**A**) **TNIL vs TL**. IPA analysis of differentially regulated SASP genes demonstrate pathways affected by these genes. Various forms of inflammation are impacted by these genes; a notable one is HMGB1 signaling that was reported to be a product of senescent placental membrane cells. (**B**) **Control vs CSE**: IPA analysis of differentially regulated SASP genes demonstrate pathways affected by these genes. Various forms of inflammation are impacted by these genes. One of the notable top 5 functions is HMGB1 signaling which was reported to be a product of senescent placental membrane cells. This pathway was seen in TNIL vs TL membranes (Figure1G).

We analyzed 12 differentially expressed genes (*TIMP1*, *IL6R*, *ICAM1*, *IL6*, *SERPINE1* [also *PAI-1*], *IL1A*, *CXCL1*, *CCL8*, *IL1B*, *CXCL8* [also *IL8*], *ANG*, and *FN1*) in TNIL and TL tissues. IPA analysis revealed that these genes represent signaling associated with inflammation, including *IL6, HMGB1, TREM1*, *IL17,* and *p53* (Figure 3A). In CSE vs control, 7 differentially expressed genes were involved in predicting various signaling mechanisms. Similar to in vivo specimens, various forms of inflammatory signaling mediated by IL6, HMGB1, TREM-1, IL17, TLR, p38 MAPK, and IL8 were predicted by differential expression of *MMP3*, *CDKN2B* (also *p15)*, *SERPINE1*, *IL6R*, *CDKN2A* (also *p16*), *PLAT*, and *CSF2* (also *GM-CSF*) (Figure 3B). This is expected in TL as well as after CSE treatment since inflammation is one of the effectors of labor and delivery as well as OS pathology associated with CSE. Besides the generalized activation of inflammation represented by genes, the top 5 canonical pathways included *HMGB1, IL6, IL17, and TREM-1* signaling, which were common between the 2 groups. All of these signaling pathways have been reported as inflammatory signals during pregnancy or in adverse pregnancy events.

The association of differentially regulated genes with HMGB1 signaling was interesting since we recently showed that this alarmin enhances placental membrane senescence and HMGB1 induces a cytokine profile characteristic of sterile inflammation [23]. The above data verify that HMGB1 signaling is likely to be operative validating our prior reports [23]. The differentially regulated genes *IL8* (*CXCL8)*, *IL1A*, *ICAM1*, and *IL1B* predict activation of HGMB1 signaling in the TNIL vs TL group, whereas the *GMCSF, IL6R, and PLAT* genes predict HMGB1 signaling induced by CSE.

**Figure 4:**
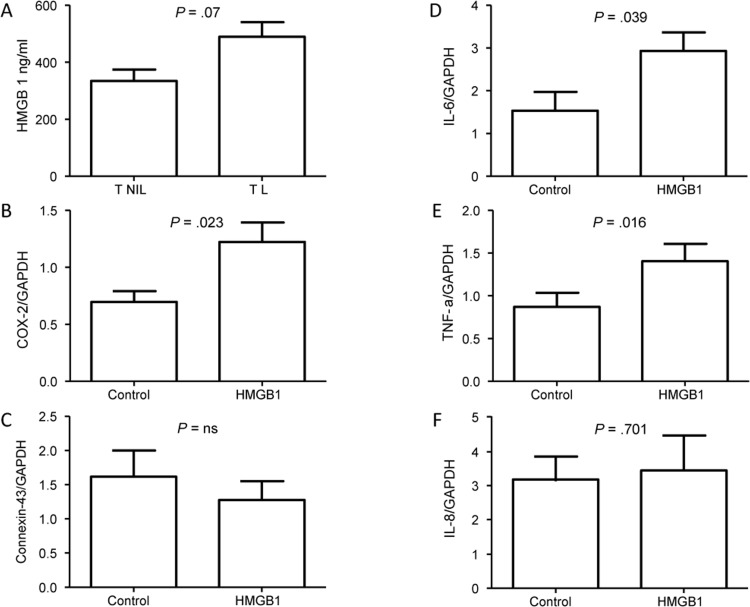
HMGB1 in placental membranes (**A**) HMGB1 concentration in the cytoplasmic fraction of placental membranes from TL was marginally higher (*P* = .07) than from TNIL. (**B**) COX-2 expression was increased in myocytes (n=6) after HMGB1 treatment compared to controls. (**C**) Connexin-43 expression was not different after HMGB1 treatment in myocytes compared to control. (**D-F**) Inflammatory cytokine genes IL-6 (**D**) and TNFα (**E**) were increased, whereas no change was seen in IL-8 expression (**F**).

Based on the bioinformatics data, we tested the idea that HMGB1 signaling is important in TL but not TNIL tissues. To determine whether senescence and cellular injury at TL cause more cytoplasmic localization of HMGB1, we measured HMGB1 in cytoplasmic fractions of TNIL and TL membranes by ELISA. HMGB1 was somewhat higher in the cytoplasmic fractions of membranes from TL (489.7 ± 102.8 ng/mL) compared to TNIL (335.0 ± 68.46 ng/mL; *P* = .07) (Figure 4A), suggesting more cellular senescence and more placental membrane tissue damage in TL and delivery than TNIL membranes.

### HMGB1 increases expression of myometrial COX-2 and inflammatory markers

To determine whether there is a functional role for HMGB1 at maternal sites after its release from senescent placental membrane cells, we measured the expression of 2 contractility-associated genes in myocytes after treatment with recombinant HMGB1. HMGB1 treatment of myometrial cells significantly increased *COX-2* (*P* = .023) (Figure 4B) but not Connexin-43 (*P* = .48) (Figure 4C) expression. Similarly, HMGB1 increased expression of the proinflammatory genes *IL6* (0.039) (Figure 4D) and TNFα (0.016) (Figure 4E) but not *IL8* expression (*P* = .7) (Figure 4F). This finding suggests that HMGB1 is a candidate mediator of at least a subset of the inflammatory signals that are needed for parturition.

## DISCUSSION

We investigated histologic and biochemical changes associated with senescence in placental membranes transitioning from not-in-labor to labor status in normal term pregnancies. We also examined the evidence for cellular senescence and SASP-associated gene expression between TNIL and TL membranes to determine the potential impact of these genes in promoting labor-related changes in the maternal myometrium. Our principal findings are as follows: 1) the TNIL to TL transition is associated with reduced telomere length in the placental membranes, as well as p38 MAPK activation, lamin B1 loss, and increased SA-β-gal positivity, which are hallmarks of senescent cells; 2) with the SASP-associated markers tested, differentially regulated genes indicated the involvement of inflammatory signaling, with signaling due to the alarmin, HMGB1 as a top candidate; and 3) cytosolic HMGB1 increased in TL samples, indicative of depletion from the nucleus and subsequent secretion. Our prior reports have shown increased release of HMGB1 into tissue culture environment [23]. These data were recapitulated in placental membranes from TNIL exposed to OS in vitro. Senescent-specific histologic and biochemical changes, as well as changes in SASP gene expression during TL and after CSE-treatment, suggest that senescence-associated inflammation, mediated in part by HMGB1 signaling, is a major pathway involved in parturition. Our cell culture data further support our hypothesis that TNIL placental membranes are progressively aging, due in part to cellular senescence, during pregnancy and that this transition can be mediated by OS. In support of this idea, recombinant HMGB1 increased contractility and inflammatory-associated gene expression in myometrial cells.

The telomere length reduction shown here prompted us to perform γ-H2AX staining. The key difference noted in our clinical samples and cell culture studies is that γ-H2AX foci, generally indicative of DNA double-strand breaks [38, 55], is not different in TL vs TNIL membranes, unlike CSE-treated amnion cells and untreated cells. Under physiologic OS, expected at term, extensive DNA damage and increased γ-H2AX foci [56,57] are not expected. However, CSE is known to cause double-strand DNA breaks, DNA damage foci, and reduced telomere lengths in children born to mothers who smoked during pregnancy [38,40,58-61]. These effects support our data which show that the extent of OS damages on DNA are likely different in TL in vivo compared to CSE-induced changes in cell cultures.

Cellular senescence and signals from injured cells have been postulated as fetal signals to initiate parturition [3]. We examined the expression of a subset of SASP genes [22, 35] in TNIL and TL and also in culture after CSE treatment. Many of these genes are involved in inflammation or regulation of the cell cycle. Bioinformatic analysis showed that the pathways in which these genes participate drive inflammation, an expected phenomenon from senescent cells [62]. We further identified HMGB1 signaling as a common pathway. As a DAMP and alarmin, HMGB1 translocates to the cytosol and is secreted after injury; outside the cell, it functions as an inflammatory cytokine [63-65]. HMGB1 is implicated in pregnancy complications, such as preterm birth and chorioamnionitis [66-68]. HMGB1 increased cellular senescence [23], myometrial contractility, and inflammatory gene expressions in our studies. We reported similar mechanisms by cell-free fetal telomere fragments, another DAMP, functioning similarly in intrauterine tissues [25]. Data from these studies indicate that the SASP and DAMPs, either independently or synergistically, increase the overall sterile inflammatory load in uterine tissues. This overload may tilt the homoeostatic balance of the uterine cavity from a quiescent to an active status with regard to contractility. SASP and DAMPs can be considered potential signals of parturition with abilities to cause labor-associated transitions.

One limitation of this study is that our in vivo results can only partially be copied by our CSE-induced OS model in culture. OS generated by the maturing fetus and antioxidant status of the uterine cavity cannot be precisely mimicked in culture by CSE or other laboratory OS inducers, such as H_2_O_2_ or glucose oxidase. Therefore, the pathways, their downstream effectors, and resulting phenotype can differ with the type of stimulant. Regardless, we recapitulated most of the in vivo findings in clinical specimens in culture. A second limitation is that we examined only 46 senescence and SASP-associated genes. A SASP signature of placental membranes or intrauterine tissues at term parturition has not been established and could differ from the reported SASP signatures in other cells or tissues. We have also not tested the effect of placental membrane senescence as a mechanism of parturition in animal models since parturition signals in animal models differ from those in humans.

**Figure 5:**
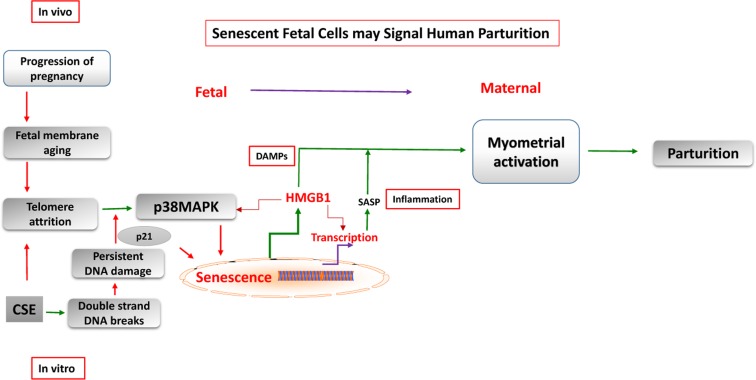
Feto-maternal signaling in parturition Senescence of human placental membranes is associated with telomere attrition and p38 MAPK activation. Senescent membranes contribute to sterile inflammation by generation of SASP and DAMPs, like HMGB1. These signals from senescent fetal cells increase the overall inflammatory load in intrauterine tissues, disrupt the homeostatic balance, and cause myometrial activation.

A classic feature of human parturition is functional progesterone withdrawal, in which maternal myometrial smooth muscles transform from quiescence status (noncontractile) to an active contractile form under inflammatory duress. This transition is mediated, in part, by switching of the progesterone receptor (PR)-A:PR-B ratio in myometrial cells from PR-B-dominance (mediates anti-inflammatory and relaxing actions of progesterone) to PR-A-dominance (inhibits the anti-inflammatory actions of progesterone and increase contractility). Here we demonstrate that the SASP and DAMP factor HMGB1 can contribute to the inflammatory overload required for the transition of myometrium. We suggest this process is associated with cellular senescence in placental membranes mediated by p38 MAPK. We conclude that placental membranes, as independent entities between mothers and fetuses, promote parturition in humans through cellular senescence-associated induction of sterile inflammation (Figure 5).

## METHODS

### Placental membrane collection

Placental membrane tissues were obtained after placental delivery from women undergoing elective repeat cesarean sections and uncomplicated pregnancies at TNIL or women with term vaginal deliveries (TL) at the John Sealy Hospital at The University of Texas Medical Branch in Galveston, USA. IRB approval as an exempt status for discarded tissues was obtained prior to collection of these samples. Demographic data were collected from patient interviews, and clinical data were abstracted from the medical records of the patients. Data collected included gestational age, maternal age, and a complete medical and obstetrical history. We excluded subjects who had prior histories of preterm birth or other pregnancy-related complications, reported smoking, had surgeries or other medical interventions during their pregnancy, used antibiotics, had a history of bacterial vaginosis, or had a positive Group B *Streptococcus* screening at 35 weeks.

### Amnion epithelial cell (AEC) culture

Placental membranes from TNIL women who underwent cesarean deliveries were dissected, and the amnion layer was peeled from choriodecidua and washed in warm saline; small pieces (0.5 cm^2^) were digested twice with trypsin (1 mg/mL) and collagenase (0.5 mg/mL) for 30 minutes at 37°C. The digestion buffer was inactivated by DMEM complete media (DMEM/F12 [Sigma-Aldrich, Saint Louis, MO, USA] supplemented with 15% fetal bovine serum [Sigma-Aldrich] and antibiotics [100 U/mL penicillin and 100 mg/mL streptomycin]), and the cells were collected by centrifugation. Cells were counted with a hemocytometer, and 1.5–2.0K cells were seeded in 10 cm culture flasks with DMEM complete media at 37°C in a humidified atmosphere containing 5% CO_2_. The purity of the epithelial cells was greater than 95%, as determined by staining with cytokeratin antibodies (Pan-Cytokeratin, Abcam, Cambridge, MA, USA, #ab80826). The culture media was replaced daily. To control for effects of replicative senescence, all experiments were performed 8–10 days after primary culture [34].

### Cell viability assay

Cell viability was quantified based on a fluorescence assay as described in our prior reports using propidium iodide (Sigma-Aldrich, #P4864) staining of cells [25]. Primary amnion cells were used for telomere length measurement and γ-H2AX staining since these experiments are not possible in nondividing organ explants.

### CSE preparation and stimulation of the placental membranes

Placental membranes from the placentas of elective repeat cesarean sections and uncomplicated pregnancies at term, not in labor (n=6) were cultured and stimulated with water-soluble CSE (1:10 dilution) as previously described [34,45,47,48]. After a preincubation period of 48 hours at 37°C in an atmosphere of 5% CO_2_, membranes were stimulated with CSE for an additional 24 hours. Tissue samples from CSE-stimulated cultures and unstimulated control were collected and stored at −80°C.

### SA-β-gal assay

The SA-β-gal activity, a senescent cell marker, was evaluated using a commercial histochemical staining assay, following the manufacturer's instructions (Senescence cells Histochemical Staining Kit; Sigma-Aldrich). Frozen sections were used for this study and the details of this protocol can be seen in reference # 19. The number of β-gal positive cells was scored by counting at least 300 cells per representative field and expressed as percentage of total cells.

### Immunohistochemical localization of p21 and senescence (loss of lamin B1)

Immunohistochemical localization of OS markers was done on paraffin-embedded sections (n = 8 in each group). Tissue samples that were previously prepared and mounted on slides underwent paraffin removal using xylene (4x for 5 minutes each) followed by rehydration through a series of graded alcohols, with a final rinse in phosphate buffered saline (PBS pH 7.4). Endogenous peroxides were quenched by soaking sections in 2 changes of 0.3% H_2_O_2_ in methanol for 5 minutes each. Prior to immunohistochemistry, sections were treated with antigen retrieval to facilitate antibody binding to antigen by placing them on a preheated chamber at 99°C for 20 minutes in Target Retrieval Solution (Dako Corporation, Carpinteria, CA. Cat. #S1699). The slides were then rinsed in 3 changes of PBS and placed into a container of Tris-Buffered Saline with Tween 20 (Signet Pathology Systems, Inc., Dedham, MA. Cat. #2380) for 5 minutes to decrease the surface tension of the slides and to facilitate coating by the reagents listed below. Using the standard protocols described previously [24,46,48], immunohistochemistry was performed using a Dako Autostainer (Dako Corporation, Carpinteria, CA), in which all of the steps were automated. Lamin B1 was localized using lamin B1 polyclonal antibody (H90, catalog no. sc-20682; Santa Cruz Biotechnology) with a dilution of 1:100, and p21 was localized using rabbit antihuman primary antibody (Santa Cruz Biotechnology #sc-397). Invitrogen (#A11034) Alexa Fluor 594 goat antimouse IgG (red) was used as the secondary antibody for in vitro experiments, whereas Fluor 488 goat antimouse IgG (Green) was used for in vivo clinical samples. The slides were then taken off the autostainer, rinsed in distilled water, and manually counterstained with Harris Hematoxylin (Fisher Scientific) for 1 minute (this is done only for 3-NT stained sections). Photomicroscopy was performed using an Olympus microscope (BX43 fluorescent, Olympus, Center Valley, PA).

Cells positive for p21, γ-H2AX, and lamin B1 loss were counted by 2 independent researchers who were blinded to the status of tissue (TL vs TNIL or CSE treated vs control). Ten randomly selected high-power fields of both amnion and chorion were examined separately, percentages of cells for each staining were calculated, and a Student *t* test was performed to semiquantiate and estimate significance.

### RNA isolation, cDNA preparation, and reverse transcription PCR

Placental membranes were disrupted with a Polytron homogenizer (Next Advanced Inc Bullet blender, Next Advanced Inc, NY, USA) using 1.0-mm ZrSiO beads (Next Advanced Inc) and Trizol reagent (Life Technologies, CA, USA). RNA was extracted from tissues using the Direct-zol RNA Mini Prep (Zymo-Research, CA, USA), according to the manufacturer's instructions. Genomic DNA contamination was removed with DNaseI treatment during RNA extraction. The quality and concentration of extracted total RNA were measured using Gen 5 Software, version 2.1 (Biotek Synergy H4 Hybrid Reader, Winooski, VT). RNA samples (0.1 mg/mL) were subjected to reverse transcription using the High-Capacity cDNA Archive Kit (Applied Biosystems, CA, USA), in accordance with the manufacturer's instructions. SASP-associated transcripts were quantified by qPCR using SensiFast Probe (Bioline) at the condition of 95°C for 7 minutes and 50 cycles of 95°C for 5 seconds and 60° C for 30 seconds. Quantification was normalized to β-actin and α-tubulin and reported as relative levels. Probes were from Roche Universal Probe Library system. Primer sets were used at 0.1μM (Table S1).

### Determination of telomere fragment length in amniochorion and concentrations in amniotic fluid

The quality and concentration of extracted DNA were determined by 260/280 nm absorbance ratio (Gen5, Epoch, Bio Tek, Winooski, VT, USA), and the relative concentration of telomere fragments was analyzed using quantitative real-time PCR (qPCR). References for the relative number of telomere fragments were generated by performing serial dilutions from a reference DNA sample to produce concentrations of DNA ranging from 20 to 0.625 ng/μL. Quadruplet (for standard curves) and triplicate (for samples) PCR reactions using 5 ng DNA for each sample were carried out in a 20 μL volume using a 2x DNA Master SYBR Green kit (Applied Biosystems [ABI], Foster City, CA, USA) on an ABI 7500 real-time PCR machine with SDS software, version 1.3.1. Primers for telomere (tel1b, 52-CGG TTT GTT TGG GTT TGG GTT TGG GTT TGG GTT TGG GTT-32; and tel2b, 52-GGC TTG CCT TAC CCT TAC CCT TAC CCT TAC CCT TAC CCT-32) were added to the final concentrations of 0.2μM. The thermal cycling profiles were as follows: 95°C for 10 minutes, followed by 20 cycles of 95°C for 5 seconds, 56°C for 10 seconds, and 72°C for 60 seconds. No template controls were included in all plate reactions. The relative number of telomere fragments in each specimen was normalized to the reference sample [2 -(ΔCt(sample) – ΔCt(control) = 2 - ΔΔCt] [69,70].

### Western blot analysis of P-p38 MAPK and p53

P-p38 MAPK and (p38 MAPK protein levels were assessed in TNIL and TL (n = 10 each) and control and CSE treated membrane in in vitro experiments (n=10) using the western blotting technique. Details of this protocol can be seen in our earlier publications [24,33,34].

### Assessment of DNA damage

(Immunofluorescence [IF] for phosphorylated [γ]-histone H2AX). In order to evaluate the activation of the DNA damage response in amnion cells, we performed IF for phosphorylated histone γ-H2AX. Cells were fixed in ethanol 95% for 10 minutes at RT and blocked for 1 hour in PBS containing 1% BSA. Primary antibody (γ-H2AX, Abcam #ab22551) was diluted in blocking buffer and incubated for 3 hours. The cells were washed, incubated with secondary antibody (Dye Light 488, Abcam #ab96875) for 20 minutes at RT, washed again, counter stained with 4′-6-diamidino-2-phenylindole (DAPI), and mounted with mount media. Images were acquired and analyzed under 40x magnification.

### Ingenuity systems pathway analysis (IPA) and knowledgebase for pathway identification

IPA analysis was used to examine whether the dysregulated SASP genes expressed in TL and TNIL placental membranes, as well as CSE-treated and control membranes, represented any specific canonical pathways associated with parturition. The gene variants that were significantly different between groups (*P* ≤ .05) were entered into the IPA analysis tool. Enrichment of focus genes in higher order disease categories were evaluated by comparing *P* values calculated by the IPA software. To derive *P* values, the IPA software uses a right-tailed Fisher Exact Test to calculate the likelihood that the association between the set of focus genes and a disease function is due to random chance. If a higher-order disease and disorder category contains 2 or more specific functions reaching statistical significance, the software displays the most statistically significant value on the y axis of the bar graph. We used the IPA software to identify canonical pathways determined by differentially regulated genes, as described in our prior reports [71,72]. Canonical pathways were determined based on the ratio between the genes uploaded into IPA based on fold change, *P* values (between TL vs T-NIL and CSE vs control), and the total number of gens in the pathway. The 12 genes filtered as significant at *P* value ≤ .05 in TNIL compared to TL or 7 genes in CSE compared to untreated controls were imported to IPA for network and functional analyses. By default, IPA applies a -log (*P* value) cutoff of 1.3, meaning that pathways with a *P* value equal to or greater than (less significant than) .05 are hidden. If a pathway has a high ratio (percentage) and a very low *P* value, it is probably associated with the data, and a large portion of the pathway may be involved or affected. This criterion was applied to select the canonical pathways impacted by dysregulated genes in our analysis.

### HMGB1 ELISA

After cytosolic and nuclear fractionation, a total of 25 μg in 100 μL of each fraction was incubated for 2 hours at 37°C in special plates precoated with HMGB1-specific capture antibodies along with a set of standards provided with the kit. After incubation, unbound standard or samples were washed away, a biotin-conjugated detection antibody was added, and the plate was incubated for another hour. The plate was then washed, an avidin-horseradish peroxidase conjugate was added, and the plate was again incubated for an hour. Unbound conjugate was then washed away, and a TMB substrate was added which resulted in color formation upon reacting with the HRP enzyme. After 20 minutes, the color development reaction was stopped, and the optical density was measured at a wavelength of 450 nm. The sample concentration of the unknown was then calculated by plotting the log of the standard concentrations and the log of the optical density read for each standard.

### Myometrial cell culture

These cells were a gift from Dr. Sam Mesiano at Case Western Reserve University, Cleveland, OH. The culture methods were based on Dr. Mesiano's prior reports [73-75]. HTERT-HMA/B cells were grown in Dulbecco's Modification of Eagle's Medium (DMEM) (phenol red free), Mediatech #17-205-CV supplemented with 10% charcoal-stripped fetal bovine serum (Sigma, #F6765), 0.5% Penicillin-Streptomycin solution (Mediatech, #30-001-CI), 2mM L-glutamine (Sigma, #G7513), 100 μg/mL G418 Sulfate (Mediatech #302-34-CR), 1 μg/mL Hygromycin B (Gibco, #10687-010), and 5 μg/mL Blaticidin S HCl (Invitrogen, #R210-01). Three hunrdred thousand cells were counted and seeded in 6-well plates. After 24 hours, the cells were treated with either 1 ng/mL rhHMGB1 (R&D, #1690-HMB) or control media. Treatments lasted for 24 hours. At 24 hours, protein and RNA was extracted using either Radioimmuno-precipitation assay buffer or TRIzol® Reagent (Ambion, #15596018).

### HMGB1 treatment of myocytes

To determine if HMGB1 can cause labor-associated changes in myocytes, recombinant HMGB1 (1 ng/mL) was used to stimulate myocytes (n=8). After 24-hour treatment, cells were frozen for mRNA analysis for contractility associated genes; *Connexin-43, COX-2*, and inflammatory genes *IL6*, *IL8*, and TNF-α. SYBR Green real-time PCR was performed by using an ABI 7500 Fast Real-Time PCR System (Applied Biosystems). We tested primer specificity by reverse transcription PCR (RT-PCR) and confirmed it by melting (dissociation) curve analysis. Glyceraldehyde-3-phosphate dehydro-genase (GAPDH), a housekeeping gene, was used as an internal control. Amplification was performed under the following conditions: denaturation for 30 seconds at 95°C followed by 40 cycles of denaturation for 15 seconds at 95°C and annealing/extension for 1 minute at 60°C. All reactions were performed in duplicate and no template controls were included in each run. The comparative 2-∆∆Ct method was used to calculate relative quantification of gene expression and data are presented as fold changes between groups.

## SUPPLEMENTARY TABLE


