# Supplementary material for: Placental membrane aging and HMGB1 signaling associated with human parturition
Source: Aging (Albany NY). 2016 Feb 4;8(2):216–29. doi: 10.18632/aging.100891 (PMC4789578; doi:10.18632/aging.100891)
Supplement: Supplementary file 1 [file aging-08-216-s001.pdf]

SUPPLEMENTARY TABLE

Table S1. Human primer sequences used for qPCR

Gene name	forward primer sequence	reverse primer sequence	UPL Probe #
ACTB	5'- CCAACCGCGAGAAGATGA -3'	5'- TCCATCACGATGCCAGTG -3'	64
TIMP1	5'- GGGCTTCACCAAGACCTACA -3'	5'- TGCAGGGGATGGATAAACAG -3'	76
IL6R	5'- GACATTCACAACATGGATGGTC -3'	5'- CTTGCCCGAACTCCTCCT -3'	1
ICAM1	5'- TAGAGACCCCGTTGCCTAAA -3'	5'- TCATACACCTTCCGGTTGTTC -3'	51
CDKN2A (*p16*)	5'- GAGCAGCATGGAGCCTTC -3'	5'- CGTAACTATTCGGTGCGTTG -3'	67
IL6	5'- GCCCAGCTATGAACTCCTTCT -3'	5'- GAAGGCAGCAGGCAACAC -3'	45
SERPINE 1 (PAI-1)	5'- CCAGCTGACAACAGGAGGAG -3'	5'- CCCATGAGCTCCTTGTACAGAT -3'	3
IL1A	5'- GGTTGAGTTTAAGCCAATCCA -3'	5'- TGCTGACCTAGGCTTGATGA -3'	6
CXCL1	5'- GCTGAACAGTGACAAATCCAAC -3'	5'- CTTCAGGAACAGCCACCAGT -3'	52
CCL8	5'-CCCTCAGGGACTTGCTCAG -3'	TCTCCAGCCTCTGGATAGGA	60
IL1B	5'- CTGTCCTGCGTGTTGAAAGA -3'	5'- TTGGGTAATTTTTGGGATCTACA -3'	78
CXCL8 (IL8)	5'- AGACAGCAGAGCACACAAGC -3'	5'- ATGGTTCCTTCCGGTGGT -3'	72
ANG	5'- GGGAGCCTGTGTTGGAAG -3'	5'- GCACGAAGACCAACAACAAA -3'	9
FN1	5'- GACGCATCACTTGCACTTCT -3'	5'- GCAGGTTTCCTCGATTATCCT -3'	1
MMP3	5'- CAAAACATATTTCTTTGTAGAGGACAA -3'	5'- TTCAGCTATTTGCTTGGGAAA -3'	36
CDKN2B (p15 isoform 2)	5'- CAACGGAGTCAACCGTTTC -3'	5'- CATCGGCGATCTAGGTTCC -3'	81
PLAT	5'- GGCAAGGTTTACACAGCACA -3'	5'- CATCAGGATTCCGGCAGT -3'	61
CSF2 (GM-CSF)	5'- TCTCAGAAATGTTTGACCTCCA -3'	5'- GCCCTTGAGCTTGGTGAG -3'	1
TUBA1A	5'- CTTCGTCTCCGCCATCAG -3'	5'- TTGCCAATCTGGACACCA -3'	58
